# Intelligent Detection Method for Wildlife Based on Deep Learning

**DOI:** 10.3390/s23249669

**Published:** 2023-12-07

**Authors:** Shuang Li, Haiyan Zhang, Fu Xu

**Affiliations:** 1 School of Information Science and Technology, Beijing Forestry University, Beijing 100083, China; lishuang98@bjfu.edu.cn (S.L.); xufu@bjfu.edu.cn (F.X.); 2 Engineering Research Center for Forestry-Oriented Intelligent Information Processing of National Forestry and Grassland Administration, Beijing 100083, China

**Keywords:** wildlife, deep learning, object detection, TMS-YOLO

## Abstract

Wildlife is an important part of natural ecosystems and protecting wildlife plays a crucial role in maintaining ecological balance. The wildlife detection method for images and videos based on deep learning can save a lot of labor costs and is of great significance and value for the monitoring and protection of wildlife. However, the complex and changing outdoor environment often leads to less than satisfactory detection results due to insufficient lighting, mutual occlusion, and blurriness. The TMS-YOLO (Takin, Monkey, and Snow Leopard-You Only Look Once) proposed in this paper is a modification of YOLOv7, specifically optimized for wildlife detection. It uses the designed O-ELAN (Optimized Efficient Layer Aggregation Networks) and O-SPPCSPC (Optimized Spatial Pyramid Pooling Combined with Cross Stage Partial Channel) modules and incorporates the CBAM (Convolutional Block Attention Module) to enhance its suitability for this task. In simple terms, O-ELAN can preserve a portion of the original features through residual structures when extracting image features, resulting in more background and animal features. However, O-ELAN may include more background information in the extracted features. Therefore, we use CBAM after the backbone to suppress background features and enhance animal features. Then, when fusing the features, we use O-SPPCSPC with fewer network layers to avoid overfitting. Comparative experiments were conducted on a self-built dataset and a Turkish wildlife dataset. The results demonstrated that the enhanced TMS-YOLO models outperformed YOLOv7 on both datasets. The mAP (mean Average Precision) of YOLOv7 on the two datasets was 90.5% and 94.6%, respectively. In contrast, the mAP of TMS-YOLO in the two datasets was 93.4% and 95%, respectively. These findings indicate that TMS-YOLO can achieve more accurate wildlife detection compared to YOLOv7.

## 1. Introduction

Wildlife is an important component of natural ecosystems and plays a crucial role in maintaining ecological balance and stability. Protecting wildlife and their habitats helps to maintain the stability and health of ecosystems [1]. As part of the ecosystem, wild animals can promote the reproduction and growth of plants and other organisms. Any drastic change in the population of any animal species in a certain area can have a significant impact on the ecosystem, leading to the disappearance of one or even several species in that area, as seen in the classic example of animal invasion [2].

However, collecting data on wildlife in the wild can be difficult due to the complex and ever-changing environment. The development of modern technology gives more choices and possibilities for wildlife monitoring. Infrared cameras are widely used in wildlife surveys due to their small impact on different habitats/environments, the ability to work continuously for 24 h, minimal interference with animals, and relatively low requirements for field workers [3,4,5]. Due to the development of computer vision, object detection is widely used in various fields. This study aims to develop one detection model based on deep learning using images of wildlife to assist in building a wildlife monitoring system, which can provide data support for wildlife conservation.

Currently, deep learning-based object detection algorithms can be divided into two-stage object detection and one-stage object detection based on whether there are candidate boxes. The former first extracts candidate boxes from the image and then obtains detection point results based on the candidate regions, which have high detection accuracy but slow detection speed, such as Faster R-CNN [6]. The latter directly calculates the detection results based on the image, which has a fast detection speed but a low detection accuracy, such as YOLOv1 [7]. Deep learning-based object detection algorithms have been successfully applied to various species such as mammals [8,9,10], birds [11,12,13], and insects [14,15,16]. Using deep learning for wildlife detection can greatly reduce manual costs and is also conducive to building wildlife monitoring systems and providing data support for decision-making regarding wildlife.

There have been studies on using deep learning for wildlife detection. Lu et al. [17] established a wildlife dataset and proposed WD-YOLO for wildlife detection. WD-YOLO has two main contributions: (1) a multiscale wildlife detection weighted path aggregation network for feature fusion was designed for multilayer feature maps extracted by the backbone, and (2) a neighborhood analysis non-maximum suppression method was proposed to address the problem of overlapping multiple targets, which can effectively eliminate redundant detection boxes.

Due to the toxicity of slow lorises and their nocturnal activity, Lei et al. [18] proposed an improved YOLOv5 algorithm for slow loris detection. First, a slow loris object detection dataset was established, and two improvement schemes based on the YOLOv5 network were tested using the dataset. The first improvement scheme introduced convolutional attention mechanisms [19] and inverse convolution operations on top of YOLOv5, effectively improving the detection performance with only 0.6 M additional parameters. The second improvement scheme added a small object detection layer on top of YOLOv5, which improved the detection performance for small objects.

Since establishing a specific wildlife detection dataset requires a lot of time and manpower, Lee et al. [20] proposed an extract-append data augmentation method to extract specific objects from a limited number of images using semantic segmentation and append the corresponding objects to a large number of arbitrary background images, generating a rich dataset of wildlife with various background images to solve the problem of lacking specific data that is difficult to obtain. The extract-append data augmentation method was used to improve the mAP by 2.2% for images of water deer and wild boar in Korea that could cause social problems.

To address images that are difficult to see, such as partially visible bodies, targets too close to the camera, or images taken in foggy weather, Wang et al. [21] used coordinate attention blocks on top of YOLOv5 to optimize feature fusion performance and used context information and species distribution models to improve detection performance. In summary, standard context features were extracted from input images to build a context memory bank, and self-attention modules were introduced to generate context features for query images. Finally, a suitability prediction model was generated using panda occurrence coordinates to exclude images with low survival suitability and improve detection recall.

Rancic et al. [22] used deep learning for the detection and counting of deer herds in northwest Serbia captured by drones. First, the training images were manually labeled and then experiments were carried out using YOLOv3 [23], YOLOv4 [24], YOLOv4-tiny, and SSD [25] in the train set. The experimental results showed that YOLOv4 had an error rate of 8.3% in counting, while YOLOv4-tiny misjudged 12 deer in counting, accounting for 7.1% of the error rate. Zhang et al. [26] proposed an improved YOLOv5s algorithm, which showed a 3.2% increase in mAP and an 11.9% increase in FPS compared to the original YOLOv5s algorithm. Roy et al. [27] proposed a real-time detection system for endangered wildlife called WilDect-YOLO. They conducted experiments on a self-built dataset of endangered wildlife and achieved the mAP of 96.89% at 59.2 FPS.

In wildlife detection, the probability of multiple animals appearing in a single image is high, and there are often cases where animals are similar to the background, making it easy to confuse them with the background. Pineda et al. [28] constructed a Japanese macaque dataset. Due to the good integration of the monkeys and background objects, foreground extraction and detection of objects of interest are the main challenges of this dataset. As for animals that are not very prominent in the background, such as white-tailed deer, which often appear in mysterious backgrounds and are often in the shadows in visible light images, Krishnan et al. achieved only a detection and classification rate of 15% [29].

The main difference between wildlife detection and general object detection is that the outdoor environment is complex and ever-changing, and images in the dataset often have problems such as insufficient lighting, mutual occlusion, and blurriness. This is also a difficulty in wildlife detection. This paper conducted research on a self-built wildlife object detection dataset and extended and improved YOLOv7 [30] to design TMS-YOLO, which is more suitable for wildlife detection. Simply put, O-ELAN retains some of the original features when extracting image features, making the extracted features more abundant. Then, CBAM is used to suppress the noise information retained by the backbone, reduce the depth of the network during feature fusion, and avoid overfitting. Meanwhile, experiments were conducted on a public wildlife dataset in Turkey to maintain generality.

The significance of this article for research is to assist in building a wildlife monitoring system for national parks using object detection technology and to provide data support for decision making related to wildlife protection. The main problem to be solved in this paper is the detection of wild animals in complex environments, especially the detection of wild animals that are similar to the background.

The main contributions of this article are as follows.

(1) A new wildlife object detection dataset was established, which includes three animals and 3878 images, providing data support for the detection of common wildlife in national parks.
(2) An optimized efficient layer aggregation network (O-ELAN) was designed, which can better extract the characteristics of wildlife.
(3) In the self-built dataset, compared to YOLOv7, TMS-YOLO’s mAP increased by 2.9%, which proves that TMS-YOLO is more suitable for wildlife detection.

## 2. Materials and Methods

### 2.1. Datasets

**Figure 1:**
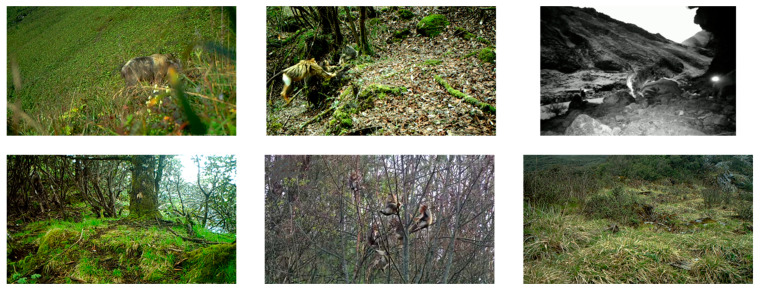
Some images in self-built dataset.

This paper resulted in the creation of a wildlife object detection dataset that includes three types of animals: *Budorcas taxicolor* (label: takin), *Rhinopithecus* (label: monkey), and *Panthera uncia* (label: leopard), with a total of 5724 instances (small: 107, medium: 1145, large: 4472) and 3796 images, including 2654 images in the training set, 762 images in the validation set, and 380 images in the test set. Some of the images in the dataset are from wildlife videos taken in national parks (1865 images, 3352 instances), some are from public datasets [31] (1267 images, 1258 instances), and others are from online documentaries [32,33,34] (664 images, 1114 instances). Some of the images are shown in Figure 1. It can be seen that there are various wild environments in the dataset, and the images themselves are also blurred, poorly illuminated, and blocked.

**Figure 2:**
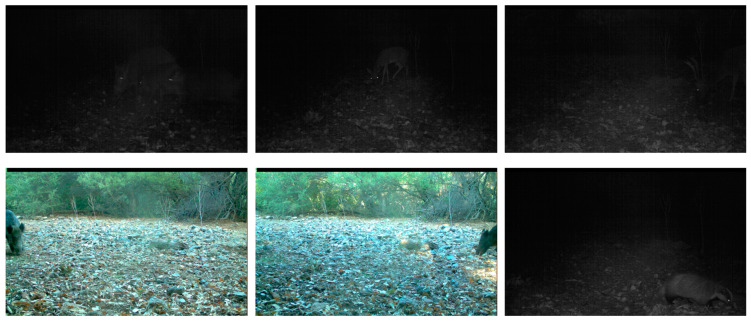
Some images in the Turkish wildlife dataset.

At the same time, a Turkish wildlife dataset [35] was downloaded, which belongs to the Ministry of Forestry and Water Affairs of the Republic of Turkey and is shared with its permission. The dataset consists of 4005 images, including various types and sizes of animals. 2585 images contain animals (some contain multiple animals), and 1420 images do not contain animals. The sizes of the images range from 1024 × 1280 to 2448 × 3264. About half of the images were taken at night and the other half were taken during the day. The dataset was divided into the training set, validation set, and test set according to the same split ratio of 7:2:1 as the self-built dataset. Some of the images are shown in Figure 2.

### 2.2. Overview of Methods

**Figure 3:**
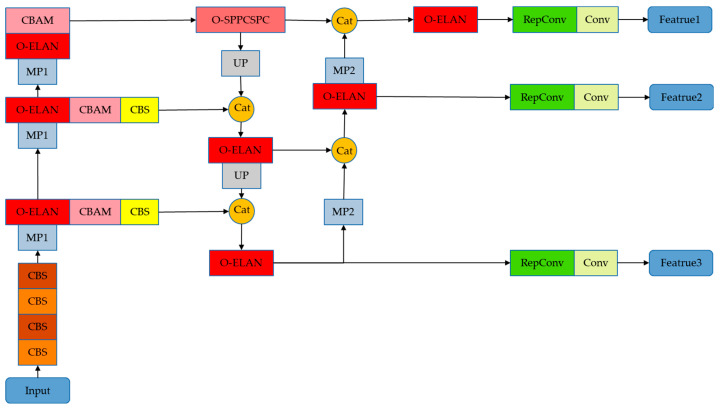
Network structure diagram of TMS-YOLO.

The TMS-YOLO proposed in this paper is based on YOLOv7, which optimizes the ELAN and SPPCSPC modules and adds CBAM. It can learn more feature information in complex field images and achieve precise detection of wildlife. Its network structure diagram is shown in Figure 3.

### 2.3. Optimized Efficient Layer Aggregation Networks, O-ELAN

ELAN enables the network to learn more features and have stronger robustness by controlling the shortest and longest gradient paths. Two ELAN structures are used in YOLOv7, one in the backbone and the other in the head.

**Figure 4:**
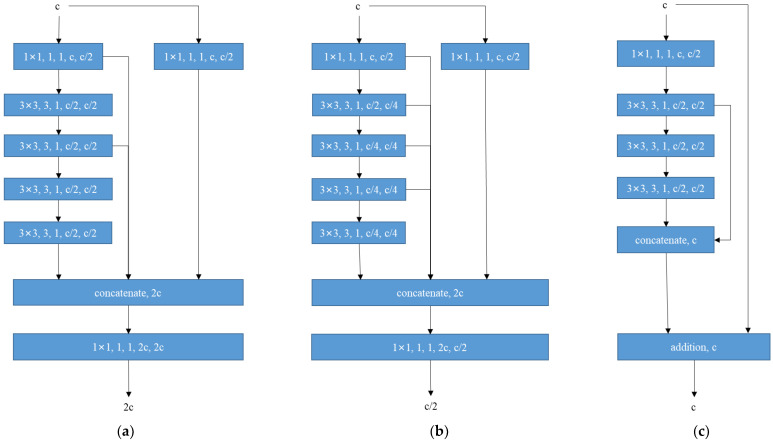
Optimized efficient layer aggregation networks; (**a**) backbone-ELAN; (**b**) head-ELAN; (**c**) O-ELAN.

The ELAN structure used in the backbone, as shown in Figure 4a, has two branches for input with c channels. One branch changes the number of channels through a 1 × 1 convolution, while the other branch is slightly more complex. After passing through a 1x1 convolution, it passes through four 3 × 3 convolutions for feature extraction. Then, it concatenates the outputs of the first, third, and fifth convolutions with the output of the first branch, resulting in an output of 2c channels. Finally, a 1 × 1 convolution is performed to change the number of channels to obtain the final result.

The ELAN structure used in the head is very similar to that used in the backbone, with the main difference being the output selected for concatenation. The backbone-ELAN selects three outputs for concatenation, while the head-ELAN selects five outputs, as shown in Figure 4b.

The O-ELAN structure designed in this paper is shown in Figure 4c. The first branch is a skip connection without processing. The second branch goes through a 1 × 1 convolution and then three 3 × 3 convolutions. It selects the output of the first 3 × 3 convolution and the last 3 × 3 convolution for concatenation, adds the concatenated result to the output of the first branch, and obtains the final output.

Due to the complexity and variability of the outdoor environment that can interfere with image recognition in wildlife detection, retaining some of the original features during feature extraction can effectively combat this interference. Therefore, the residual connection structure of O-ELAN is better suited for the task of detecting wildlife. Additionally, to extract features effectively, a simple structure similar to ELAN was designed in another branch of the residual structure. With these two branches complementing each other, better features can be extracted from wildlife images.

In the dataset used in this article, for example, the *Rhinopithecus* in the upper left corner of Figure 4a is easily overlooked. However, the O-ELAN skip connection can preserve some of the original input features, making it more advantageous when processing wildlife images. To visually demonstrate the advantages of O-ELAN, we visualized the features extracted from three ELAN structures.

**Figure 5:**
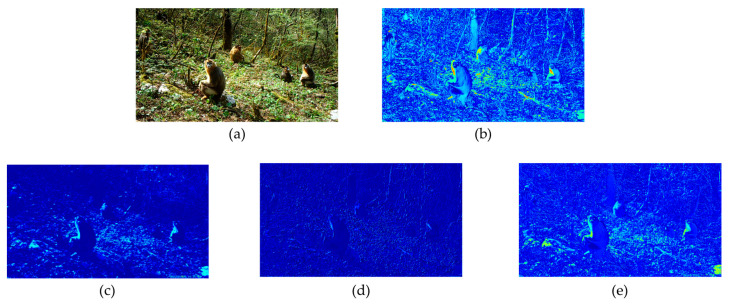
Results of ELAN. Since ELAN has requirements for the number of channels, (**a**) is first modified by convolution to adjust the number of channels. The results of (**c**), (**d**), and (**e**) are all based on the input of (**b**). (**c**) shows the features extracted by backbone-ELAN, Figure 5d shows the features extracted by head-ELAN, and Figure 5e shows the features extracted by O-ELAN.

The results of the three ELAN structures are shown in Figure 5. It is obvious that the features extracted by O-ELAN contain more information and have the best performance.

### 2.4. Optimized Spatial Pyramid Pooling Combined with Cross Stage Partial Channel, O-SPPCSPC

**Figure 6:**
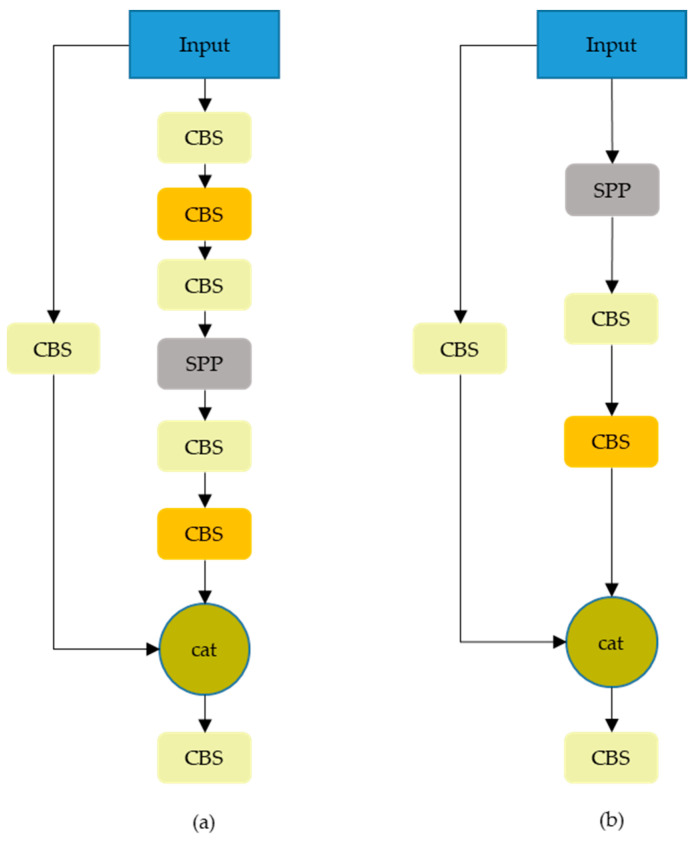
Structures of SPPCSPC and O-SPPCSPC. (**a**) is the SPPCSPC and (**b**) is the O-SPPCSPC.

The SPPCSPC in YOLOv7 combines the feature map before the SPP (Spatial Pyramid Pooling) module with the SPP module, which enriches the feature information. This paper improves the SPPCSPC module, and TMS-YOLO removes the convolution that enters the SPP. As shown in Figure 6, (a) is the SPPCSPC and (b) is the O-SPPCSPC. When the network is too deep, subtle features of the background are prone to overfitting, such as wildlife features. Therefore, we have removed some network layers before entering the SPP to reduce the occurrence of this situation.

**Figure 7:**
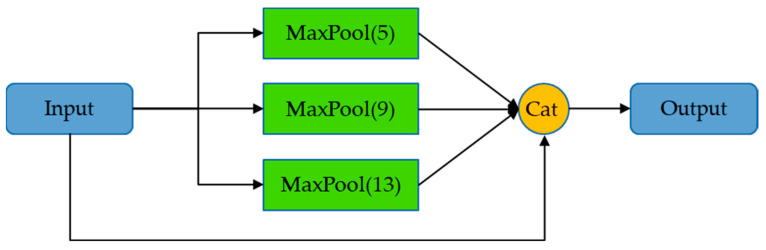
Structure of SPP.

The SPP module is shown in Figure 7, which obtains different receptive fields through max-pooling to increase the receptive field and make the algorithm adapt to images of different resolutions. As can be seen, there are four branches in the SPP, one of which is not processed, and the other three perform max-pooling of 5, 9, and 13, respectively, and then combine the results. In this way, the model can distinguish targets of different scales through different receptive fields. The two branches of O-SPPCSPC combine conventional processing and SPP processing, which can avoid image distortion caused by cropping and scaling operations on image regions and reduce half of the calculation.

### 2.5. Convolutional Block Attention Module, CBAM

**Figure 8:**
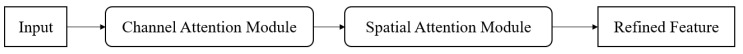
CBAM.

The attention mechanism in neural networks is a resource allocation scheme that allocates computing resources to more important tasks while solving the problem of information overload when computing power is limited. In neural network learning, by introducing attention mechanisms, it is possible to focus on the information that is more critical to the current task among a large amount of input information, reduce the attention paid to other information, and even filter out irrelevant information, which can solve the problem of information overload and improve the efficiency and accuracy of task processing. Generally speaking, the mechanisms of attention can be divided into spatial attention, channel attention, and mixed attention. As shown in Figure 8, CBAM is a mixed attention mechanism.

**Figure 9:**
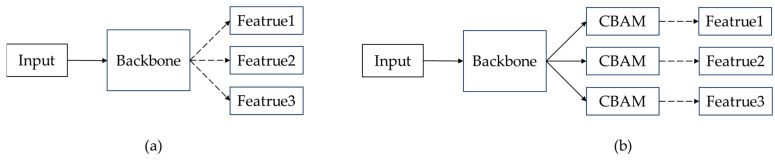
Position of CBAM in TMS-YOLO. (**a**) is the backbone of YOLOv7, which directly obtains three feature maps; (**b**) is the backbone of TMS-YOLO, which must pass through the CBAM module.

In this paper, in TMS-YOLO, CBAM is added after the three feature maps output by the backbone to optimize the feature maps output by the backbone and improve the detection effect. O-ELAN retains some original features during feature extraction, which can better extract wildlife features, but also introduces some noise information from the background. Therefore, after feature extraction by the backbone, it is necessary to use CBAM to further suppress noise information. As shown in Figure 9.

### 2.6. Experimental Parameters

Using a local server, we built a Pytorch 1.11.0 deep learning framework with the Ubuntu 20.04 operating system, CUDA version 11.3, and an NVIDIA TITAN RTX graphics card with 24 G of memory. We used the Adam optimizer with an initial learning rate of 0.001, an image input size of 640 × 640, and mixed precision training.

We set a 50% chance of performing mosaic data augmentation at each step and a 50% chance of performing mixup data augmentation after the mosaic data augmentation. As the training images generated by the mosaic data augmentation are far from the real distribution of natural images, we only performed mosaic data augmentation in the first 70% of the epochs. We used the Adam optimizer and saved the weights every 5 epochs. We trained a total of 100 epochs in the self-built wildlife dataset and we trained a total of 150 epochs in the Turkish wildlife dataset.

### 2.7. Evaluation Metrics

**Figure 10:**
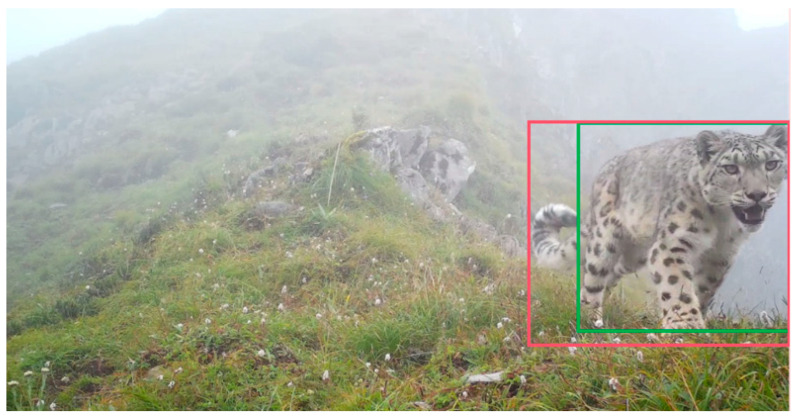
IoU. The green box is the labeled box and the red box is the predicted box.

As shown in Figure 10, the IoU value is the ratio between the area of the intersection of the red and green boxes and the area of the concatenated boxes. Generally speaking, the larger the IoU value, the better the detection effect. The predicted positive sample is denoted as P, otherwise, it is denoted as N. The predicted value is the same as the true value, and it is denoted as T, otherwise, it is denoted as F.

Precision is the number of predicted positive samples divided by the number of predicted samples, as shown in Equation (1).

(1) $$\mathrm{precision}=\frac{TP}{TP+FP}$$

Recall is the number of predicted positive samples divided by the number of true positive samples, as shown in Equation (2).

(2) $$\mathrm{recall}=\frac{TP}{TP+FN}$$

The factors for Equations (1) and (2) are explained in Table 1.

**Figure 11:**
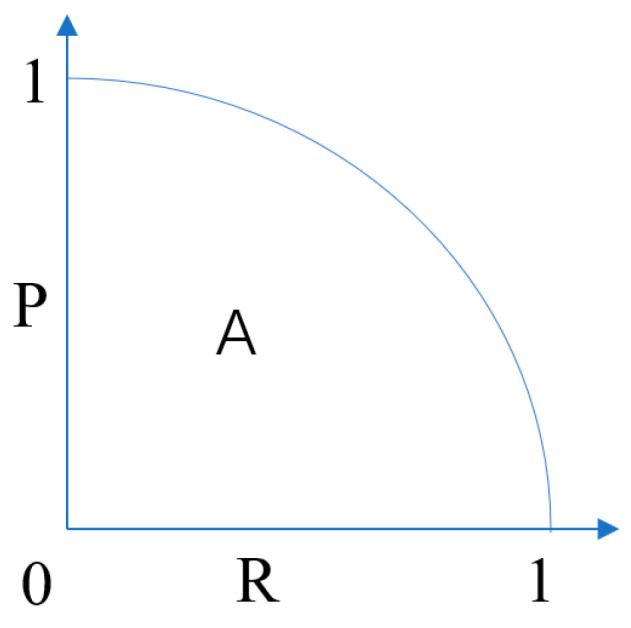
PR curve.

AP (Average Precision) is used to represent the average accuracy of detection at different recall rates and is an evaluation of the effectiveness of a target detector for a specific class. It consists of a P-R curve and an area enclosed by coordinates, as shown in Figure 11, where P represents precision, R represents recall, and the size of the area in the A part represents the PR value.

mAP is the average value of AP for each category, as shown in Equation (3), which is used to average the effect of detection for all categories of objects and is the final measure of the detection performance.

(3) $$\mathrm{mAP}=\frac{1}{n}\sum_{1}^{n}\mathrm{AP}$$

## 3. Results

### 3.1. Experimental Results on the Self-Built Dataset

#### 3.1.1. Analysis of Results

The experimental results on the self-built dataset are shown in Table 2. It is evident that TMS-YOLO outperforms YOLOv7 in all metrics, and it surpasses YOLOv8 in the most important metric, mAP. AP-L is the Average Precision calculated for large instances, respectively. Because the number of large instances accounts for 80% of the dataset, we only calculated its AP. Obviously TMS-YOLO performs the best for the large instances. This indicates that it is more suitable for wildlife detection in national parks.

**Figure 12:**
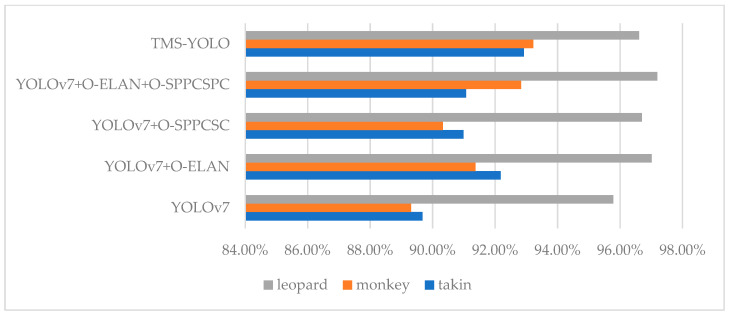
AP of models on each label.

For the AP of the models on each label, see Figure 12. On the basis of the results, the metrics for leopard are significantly better than those of takin and monkey. This is because a majority of leopard images come from a publicly available dataset with a simpler background, making it easier for the model to learn its features. On the other hand, monkey and takin have lower metrics due to complex image backgrounds and frequent occlusions, which makes it more challenging for the model to perform well. In general, TMS-YOLO shows the most significant improvement in detecting monkeys, which had the worst performance in YOLOv7. This indicates that TMS-YOLO performs better when faced with issues such as blurriness and occlusions that are commonly found in wildlife images.

#### 3.1.2. Partial Detection Results

**Figure 13:**
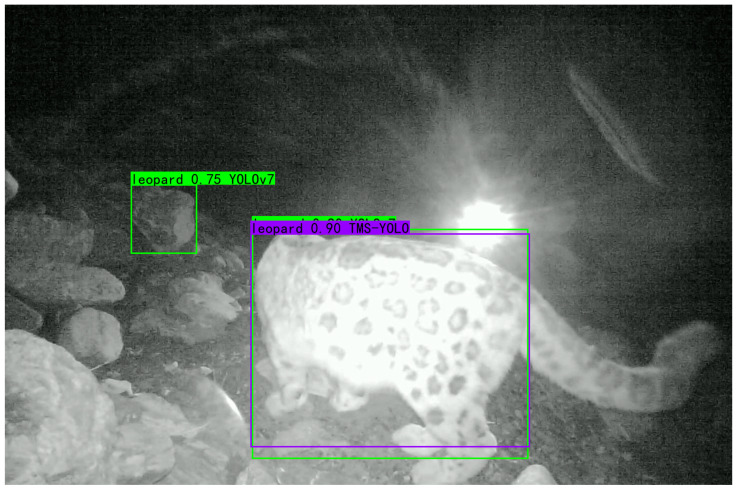
One detection result on self-built dataset. The green boxes are the predictions of YOLOv7, and the purple boxes are the prediction of TMS-YOLO.

**Figure 14:**
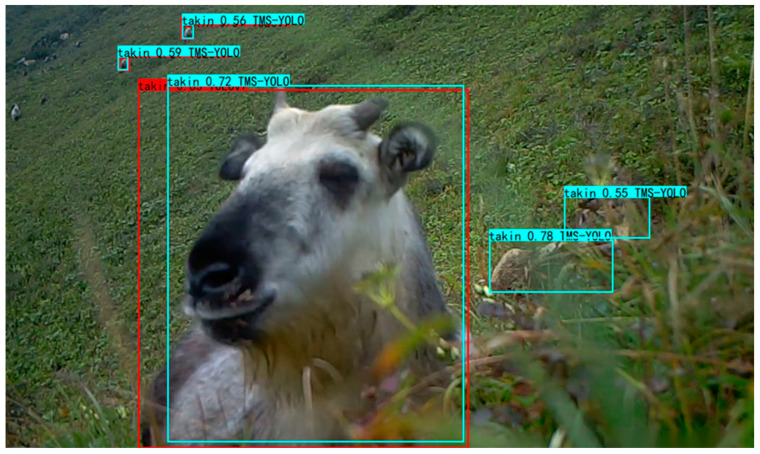
One detection result on self-built dataset. The red boxes are the predictions of YOLOv7, and the cyan boxes are the predictions of TMS-YOLO.

Some of the detection results are shown in Figure 13 and Figure 14, the green and red boxes are YOLOv7 predictions, and the purple and cyan boxes are TMS-YOLO predictions. For Figure 13, YOLOv7 mistakenly classified the background as a leopard, possibly due to a similar shape and color. For Figure 14, YOLOv7 missed the takin on the right side, whereas TMS-YOLO did not. In conclusion, TMS-YOLO demonstrates higher accuracy in wildlife detection.

### 3.2. Experimental Results on Turkish Wildlife Dataset

**Figure 15:**
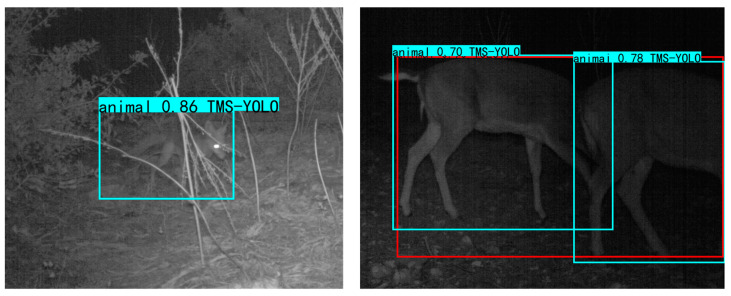
Partial detection results on Turkish Wildlife Dataset. The red boxes are the predictions of YOLOv7, and the cyan boxes are the predictions of TMS-YOLO.

The experimental results on the Turkish Wildlife dataset show that the mAP of TMS-YOLO is 0.4% higher than YOLOv7, reaching 95%. This further demonstrates that the designed improvements are effective and the structure of TMS-YOLO can improve the detection effect, which helps monitor wildlife. Some of the detection results are shown in Figure 15, TMS-YOLO performs much better than YOLOv7 in detecting low light levels at night.

## 4. Discussion

To achieve accurate detection of wildlife, the TMS-YOLO proposed in this paper is based on YOLOv7, which optimizes the ELAN and SPPCSPC modules, and adds CBAM to make it more suitable for wildlife detection.

From the experimental results in Table 2, it can be confirmed that the designs of O-ELAN and O-SPPCSPC are effective, improving the mAP by 2% and 1.2% in the self-built dataset, respectively. Moreover, TMS-YOLO achieved a 2.9% higher mAP on the self-built dataset than YOLOv7, and a 0.5% higher mAP than YOLOv8. Therefore, TMS-YOLO is effective and better at detecting wildlife.

In terms of the detection results, YOLOv7 had more cases of missed and false detection, and even detected one animal as two different animals. Although TMS-YOLO also had cases of missed detection, its false detection was better than YOLOv7.

In summary, the designed TMS-YOLO has improved the detection performance based on YOLOv7 and has significantly improved the detection effect for wildlife.

## 5. Conclusions

This study aims to develop one deep learning model using images of wildlife to assist in building a wildlife monitoring system. This article presents improvements made to YOLOv7 for the automatic detection of animals captured by cameras. A dataset was created that includes 3 types of animals (*Budorcas taxicolor*, *Rhinopithecus*, and *Panthera uncia*). Furthermore, the algorithm was tested using a Turkish wildlife dataset, which proved its suitability to detect wildlife. This work will aid wildlife researchers in monitoring the activities of the three animal species throughout national parks.

**Figure 16:**
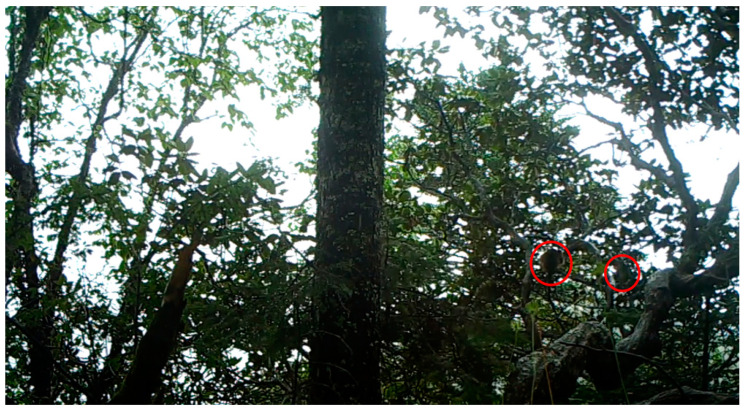
Two hard-to-detect *Rhinopithecus*. Because they are similar to the branches of the tree, these two *Rhinopithecus* inside the red box are difficult to detect.

Our method improves detection performance from the perspective of model optimization. Compared to YOLOv7, the main improvements of TMS-YOLO are to improve the detection effect of wildlife that are similar in color to the background, such as *Rhinopithecus*. However, there are still shortcomings, as seen in the example of *Rhinopithecus* climbing and jumping on trees, which makes it difficult to distinguish their bodies from tree branches when they are far from the camera and the image is blurry, as shown in the red circle of Figure 16. Future improvements can be made by increasing the resolution of the images and merging semantic information to further improve detection performance. Based on image detection, high-precision automatic video detection can also be achieved by adding pre-frame and post-frame images to videos.

## Figures and Tables

**Table 1:** The explication of the factors.

Factor	Explication
TP	the number of samples predicted to be positive and the same as the true value
FP	the number of samples predicted to be positive but different from the true value
FN	the number of samples predicted to be negative but different from the true value

**Table 2:** Result of the self-built dataset.

Model	mAP	AP-L@0.5
YOLOv7	90.5%	95.8%
YOLOv5	88.5%	97%
YOLOv6	87%	95.5%
YOLOv8	92.9%	96.7%
YOLOv7+O-ELAN	92.5%	96.9%
YOLOv7+O-SPPCSPC	91.7%	96.4%
YOLOv7+O-ELAN+O-SPPCSPC	92.8%	97.5%
YOLOv7+O-ELAN+O-SPPCSPC+CBAM(TMS-YOLO)	93.4%	97.6%

## Data Availability

The species name and images in the Türkiye Wildlife Dataset can be downloaded at: https://aistudio.baidu.com/aistudio/datasetdetail/180216 (accessed on 2 May 2023).
